# Pain relief and cartilage repair by Nanofat against osteoarthritis: preclinical and clinical evidence

**DOI:** 10.1186/s13287-021-02538-9

**Published:** 2021-08-26

**Authors:** Zuxiang Chen, Yanzhi Ge, Li Zhou, Ting Li, Bo Yan, Junjie Chen, Jiefeng Huang, Wenxi Du, Shuaijie Lv, Peijian Tong, Letian Shan

**Affiliations:** 1 The First Affiliated Hospital, Zhejiang Chinese Medical University, Hangzhou, 310053 Zhejiang People’s Republic of China; 2 The First Affiliated Hospital, College of Medicine, Zhejiang University, Hangzhou, 310003 Zhejiang People’s Republic of China; 3 Cell Resource Bank and Integrated Cell Preparation Center of Xiaoshan District, Hangzhou Regional Cell Preparation Center (Sanjiang Shangyu Biotechnology Co., Ltd), Hangzhou, People’s Republic of China

**Keywords:** Nanofat, Adipose tissue-derived stem cells, Osteoarthritis, Paracrine, Conditioned medium

## Abstract

**Background:**

Osteoarthritis (OA) is the most common joint degenerative disorder, with little effective therapy to date. Nanofat is a cocktail of cells obtained from fat tissue, which possesses regenerative capacity and has a potential in treating OA. This study aimed to determine the anti-OA efficacy of Nanofat from basic and clinical aspects and explore its action mode.

**Methods:**

Flow cytometry was performed to characterize Nanofat. A monoiodoacetate-induced OA rat model was employed for in vivo study. Cell viability and wound healing assays were conducted for in vitro study. Real-time PCR and Western blot assays were applied to explore the molecular action mode of Nanofat. Moreover, a retrospective analysis was conducted to determine the clinical efficacy and safety of Nanofat on knee OA patients.

**Results:**

The in vivo results showed that Nanofat significantly attenuated pain symptoms and protected cartilage ECM (Col2) from damage, and its effects were not significantly differed with adipose tissue-derived stem cells (both *P* > 0.05). The in vitro results showed that Nanofat promoted the cell viability and migration of chondrocytes and significantly restored the IL-1β-induced abnormal gene expressions of *Col2*, *Aggrecan*, *Sox9*, *Adamts5*, *Mmp3*, *Mmp9 Mmp13*, *IL-6* and *Col10* and protein expressions of Col2, MMP9, MMP13, and Sox9 of chondrocytes. The regulatory actions of Nanofat on these anabolic, catabolic, and hypertrophic molecules of chondrocytes were similar between two treatment routes: co-culture and conditioned medium, suggesting a paracrine-based mode of action of Nanofat. Moreover, the clinical data showed that Nanofat relieved pain and repaired damaged cartilage of OA patients, with no adverse events.

**Conclusion:**

In sum, this study demonstrated the anti-OA efficacy as well as a paracrine-based action mode of Nanofat, providing novel knowledge of Nanofat and suggesting it as a promising and practical cell therapy for clinical treatment of OA.

## Introduction

Osteoarthritis (OA) is the most common joint degenerative disorder, affecting hundreds of thousands of patients around the world, especially the middle-aged and old people [1]. Approximately 15% of the global population suffers from OA, which accounts for 2.4% of all disabled people worldwide. Aging, obesity and joint trauma are risk factors contributing to the occurrence and development of OA [2, 3]. The high prevalence of these factors brings serious harm to human beings and places huge economic burden on society [4]. Joint pain, cartilage destruction and synovial inflammation are the primary symptoms of this disease, and the inflammatory damage of articular chondrocytes and the progressive degradation of chondrocyte extracellular matrix play key roles in the pathogenesis of this disease [5]. Aiming at the OA-induced pathological changes, various treatments have been developed. Pharmaceutical treatment is a conventional strategy for OA before surgery, applying nonsteroidal anti-inflammatory drugs (NSAIDs), opioids, local analgesics, corticosteroid injection and hyaluronic acid injection [6]. However, these treatments are usually palliative and merely provide symptomatic relief from pain, failing to prevent cartilage damage and subsequent destruction of other joint tissues [7]. Moreover, many of them have side effects and poor efficacy, e.g., corticosteroid injection may lead to further joint degradation, and the analgesic effects of hyaluronic acid are controversial [8]. In sum, current nonsurgical treatments cannot satisfy the clinical needs, resulting in OA progression and unavoidable surgery on patients [9]. Thus, it is urgently needed to develop new therapeutics with better effectiveness and safety.

Cell therapy-based approaches, such as platelet-rich plasma (PRP) and mesenchymal stem cells (MSCs), have attracted increasing attention due to their unique bioactivities and potential in regenerative medicine [10]. Platelet-rich plasma (PRP) is an autologous platelet concentrate produced by one- or two-stage centrifugation procedure, releasing growth factors through activation of α-granules in platelets [11]. Ascribing to the released growth factors, such as platelet-derived growth factor (PDGF), tumor-like growth factor-β (TGF-β), fibroblast growth factor (FGF), vascular endothelial growth factor (VEGF), hepatocyte growth factor (HGF) and insulin-like growth factor-1 (IGF-1), PRP has therapeutic potential in cartilage repair [12]. However, the preparation of PRP lacks a uniform standard, resulting in uncontrolled quality that negatively affects its effectiveness [13]. MSCs are a broad category of adult multipotent stromal cells isolated from adult tissues, possessing self-renewal capacity, multilineage differentiation potential, paracrine effects and immunomodulatory properties [14]. Previous studies have applied MSCs to treat OA and observed positive outcome [15]. However, the clinical use of MSCs has raised ethical concerns: (1) in vitro expansion of MSCs may induce genetic and epigenetic changes; (2) lack of identification criteria and specific markers for MSCs; (3) vague definition of MSCs; and (4) heteroplasia and tumorigenesis risk [16–19]. Moreover, the in vitro MSCs expansion has potential clinical risk of delayed treatment because it costs time. Therefore, the clinical applications of MSCs have been strictly restricted worldwide, unless MSCs can be developed and registered as cell drug [20]. To date, except for clinical trial, there is no legal way to clinically apply MSCs in many countries. Due to the above shortcomings of PRP and MSCs, other cell therapies with better outcomes should be focused on and investigated for OA treatment.

Nanofat, also named stromal vascular fraction, is a cocktail of cells obtained from fat tissue through mechanical/enzymatic digestion, centrifugation and filtration [21]. Nanofat is devoid of adipocytes and consists of a heterogeneous population of MSCs, endothelial progenitor cells (EPCs), pericytes, stromal cells and immune cells [22]. There are several advantages of Nanofat over MSCs and other cell therapies: (1) Nanofat can be directly and readily obtained from fat tissue without overnight cell culturing preparation, making it comparatively safer than MSCs with fewer restrictions [21]; (2) the different types of cells in Nanofat have a synergistic effect on immunomodulation, anti-inflammatory, angiogenesis, and so forth, which may exert better efficacy than single-cell therapy [23]; and (3) the culture expansions lead to loss of homing property of MSC [24], possibly resulting in the homing ability of Nanofat stronger than that of culture-expanded MSC. Recently, Nanofat has been applied in regenerative medicine for skin repair [25], hair growth [26], and bone regeneration [21]. Clinically, intra-articular injection of Nanofat has exhibited promising potential in OA management, in which the WOMAC score and VAS score of OA patients were significantly improved without any adverse events [27]. However, to date, the evidence of anti-OA efficacy of Nanofat is still insufficient, and its mechanism of action remains unclear. Whether Nanofat can protect cartilage and chondrocytes from OA damage, or whether it can inhibit OA-induced inflammatory processes, requires further investigation.

In the present study, we evaluated the anti-OA efficacy of Nanofat by assessing joint pain, cartilage histology and cartilage immunohistochemistry on monoiodoacetate (MIA)-induced OA rat model. Considering MSCs as the main active component of Nanofat, we compared the therapeutic effect of Nanofat with adipose-derived MSCs (ADSCs). Since various cell types are contained by Nanofat, many soluble factors can be secreted, possibly resulting in a paracrine outcome. Accordingly, we applied Nanofat-conditioned medium (Nanofat-CM) to evaluate the paracrine-dependent action mode of Nanofat. Moreover, a retrospective study was conducted to verify the clinical efficacy of Nanofat on OA patients. This is the first study combining animal experiment and clinical trial for the study of Nanofat and exploring the paracrine-dependent action mode of this cell therapy, providing a promising therapeutic strategy for anti-OA treatment.

## Materials and methods

### Reagents

Minimum essential medium-alpha modification (α-MEM) with GlutaMAX™-1 was purchased from Gibco BRL (NY, USA). IMDM (Iscove’s modified Dulbecco’s medium) and trypsin (0.25%) were purchased from Thermo Fisher Scientific (MA, USA). Fetal bovine serum (FBS) was purchased from CellMax (Beijing, China). Cell culture plates were purchased from Eppendorf (Hamburg, Germany), and Transwell chambers were purchased from Corning (NY, USA). MIA was purchased from Sigma-Aldrich. Real-time PCR (polymerase chain reaction) kit and TRIzol reagent were purchased from TaKaRa Biotechnology Co. Ltd. (Dalian, China). In situ cell death detection TUNEL kit was purchased from Roche (Indianapolis, USA). All-in-One cDNA Synthesis SuperMix kit was purchased from BioTool (TX, USA). 2 × SYBR Green qPCR Master Mix (low ROX) kit was obtained from Bimake (TX, USA). All primary antibodies were purchased from Cell Signaling Technology Inc. (Danvers, MA, USA).

### Preparation of Nanofat and ADSCs

Collagenase digestion was used for rat ADSCs and Nanofat extraction according to the methods of previous reports [28]. Briefly, rats were anesthetized by 3% pentobarbital and their inguinal adipose tissue was collected under aseptic conditions and placed in petri dishes containing Hank’s balanced salt solution (HBSS). The blood, blood vessels and fascia were removed, and the tissue was washed repeatedly 3–5 times in HBSS. The cleaned adipose tissue was cut into pieces and placed into a 15-ml centrifuge tube, then digested with 0.2% collagenase type IV and centrifuged for 45 min at 37 °C. After digestion, HBSS was added to stop digestion and the top lipid layer was removed by centrifugation at 1000 r/min for 5 min. The cells were resuspended and filtered with a 100-μm cell strainer and then washed by centrifugation at 1000 r/min for 5 min. The supernatant was discarded, and the spun-down heterogeneous cells were used as Nanofat. For isolation of ADSCs, an aliquot of the precipitate was incubated in plates with α-MEM containing 10% FBS at 37 °C and 5% CO_2_. After 1-h incubation, the plates were washed by PBS to remove nonadherent cells, and the residual cells were subsequently cultured for 1 week with daily change of medium to obtain ADSCs.

### Characterization of Nanofat by flow cytometry

Nanofat and ADSCs were suspended in PBS at a density of 10^6^ cells/ml and incubated with the following antibodies: fluorescein isothiocyanate (FITC)-conjugated anti-CD29, phycoerythrin (PE)-conjugated anti-CD34, Allophycocyanin (APC)-conjugated anti-CD44 and fluorescein isothiocyanate (FITC)-conjugated anti-CD90. After incubation for 30 min at room temperature, each cell suspension was centrifuged at 2000 rpm for 5 min. The supernatant was removed, and 100 μl PBS was added to resuspend the cell pellet for flow cytometry analysis (BD FACSVerse, NJ, USA).

### Animals and OA modeling

Male Sprague–Dawley (SD) rats (Grade SPF II) with a body weight of 200 ± 20 g were provided by Shanghai Super B&K Laboratory Animal Co. Ltd. (Certificate number: SCXK (Shanghai) 2018-0006). All rats were housed in cages under a pathogen-free condition with a 12-h light/dark cycle and provided with food and water ad libitum. The animal experiments were following the China legislation on the use and care of laboratory animals and approved by the Medical Norms and Ethics Committee of Zhejiang Chinese Medical University. OA model was established by applying monoiodoacetate (MIA) method as described previously. Briefly, rats were intra-articularly injected with 50 μl of 30 mg/ml MIA for 7 days, followed by treatment.

### Animal experiment

To investigate the in vivo efficacy of Nanofat and made a comparison between Nanofat and ADSCs, a total of 40 rats were employed and grouped as follows: NC as normal control group, Model as OA model group, Nanofat as Nanofat-treated model group and ADSCs as ADSC-treated model group. After the OA modeling for 1 week, rats in the NC group were intra-articularly injected with 50 μl of saline, while Nanofat group and ADSCs group were intra-articularly treated with 50 μl of Nanofat (10^6^ cells/ml) and ADSCs (10^6^ cells/ml), respectively. All treatments were weekly conducted for 4 weeks.

### Pain behavior evaluations

The mechanical withdrawal threshold (MWT) and thermal withdrawal latency (TWL) were measured by Electronic tenderness tester (Huaibei Zhenghua, China) and Plantar Test apparatus (Ugo Basile, Italy), respectively. All rats were individually placed in elevated plastic cages with wire mesh bases and given 30 min for acclimatization to the testing environment. The room temperature and humidity were kept stable throughout the experimental period. For the test of MWT, the sensing probe of electronic tenderness tester was pressed perpendicularly against the midplantar surface of the left and right hind paws of each rat more than three times. For the test of TWL, a focused beam of radiant heat (up to 35 °C) was irradiated to the plantar surface of the hind paws more than three times and held for maximal 20 s. A positive response for each test was regarded as the sharp withdrawal of the paw and paw licking.

### Histopathological and immunohistochemical analyses

All rats were killed after 4-week treatment. The joints of each rat were sampled and fixed with formalin (10%) for 24 h and decalcified with EDTA (10%) in PBS for 8 weeks. Then, each sample was embedded in paraffin and sectioned into 2–3 μm, followed by staining with HE (hematoxylin and eosin) or SO (Safranin O/Fast green). The grade of OA progression was evaluated by double-blind observation, according to OARSI scoring systems. Immunohistochemistry assay was applied to detect the expression of collagenase type II (Col2). Replicates of sample sections were incubated with 0.01 mol/l citrate buffer (pH 6.0, Solarbio, Beijing, China) at 60 °C for 4 h as antigen retrieval and then incubated overnight at 4 °C with 100 μl PBS-diluted (1:100) primary antibody against rat Col2 (rabbit anti-Col2 monoclonal antibody), Col10 (rabbit anti-Col10 monoclonal antibody), and MMP13 (mouse anti-MMP13 monoclonal antibody). After PBS wash, all sections were incubated with Horseradish peroxidase-conjugated secondary antibody (PV-9001 for Col2 and Col10, and PV-9002 for MMP13) (ZSGQ-BIO, Beijing, China) for 20 min at room temperature, followed by colorimetric detection using 3,3′-diaminobenzidine (DAB) substrate chromogen for 8 min. The immunoreactivity of Col2 was semiquantified by using Image-Pro Plus 6.0 software (Media Cybernetics, Bethesda, MD, USA) under a light microscope (NIKON 80i, Tokyo, Japan). The immunoreactivity of Col2, Col10 and MMP13 was semiquantified by using Image-Pro Plus 6.0 software (Media Cybernetics, Bethesda, MD, USA) under a light microscope (NIKON 80i, Tokyo, Japan). The number of positive cells was measured for quantifying the expression of Col10 and MMP13, and the positive area was measured for quantifying the expression of Col2. The final results were expressed as the percentage of antigen-positive area/cells to total area/cells in the selected fields.

### TUNEL assay

TUNEL (terminal deoxyribonucleotidyl transferase (TdT)-mediated biotin-16-dUTP nick-end labelling) assay was employed to observe the apoptosis of chondrocytes, following the manufacturer’s instructions. Briefly, the tissues were permeabilized with the proteinase K solution at 37 °C for 20 min, followed by incubation with the TUNEL detection liquid at 37 °C for 1 h. After washing with PBS for three times, DAPI was added to stain nuclei, and the tissues were examined via a fluorescence microscope (Carl Zeiss). The rate of apoptotic cells was quantified in three randomly selected fields of view by using three slides from each sample.

### Primary chondrocytes preparation

Primary chondrocytes were isolated from allogeneic male SD rat donors as previously described [29]. Briefly, articular cartilage tissues from rat donors were harvested and sliced into small pieces. The pieces were digested with 0.25% trypsin for 40 min at 37 °C and then treated with 0.1% collagenase II for 4 h at 37 °C. Isolated cells were filtered through a cell strainer (70 μm) and collected as chondrocytes. IMDM medium containing 10% FBS was used to culture the chondrocytes. Chondrocytes at 3 generation were used for the subsequent experiments.

### Conditioned medium preparation

Conditioned medium (CM) of Nanofat and chondrocytes was prepared for cellular experiments. Nanofat cells were seeded at a density of 50, 100, 150, 200 × 10^4^ cells/dish, respectively, and chondrocytes were seeded at a density of 100 × 10^4^ cells/dish into 10-cm dish with IMDM containing 10% FBS (fetal bovine serum) at 37 °C under 5% CO_2_. After incubation for 48 h, the cell medium was collected as CM and centrifuged at 1500 rpm for 10 min to remove cell debris. After sterile filtration through a 0.22-μm filter, the Nanofat CM and chondrocyte CM were obtained and stored at − 80 °C for further use.

### Cellular experiments

Two experimental systems, respectively, applying CM-used and a co-culture nested system, were employed to investigate the in vitro effects of Nanofat on chondrocytes. In the CM-used system, chondrocytes were divided into three groups as follows: control group, IL-1β group and Nanofat-CM group. The IL-1β group and Nanofat-CM group were modeled by pre-treating IL-1β (10 ng/ml) for 24 h. Subsequently, the Nanofat-CM group was treated with Nanofat-CM for 24 h, while the control group and IL-1β group were treated with chondrocyte CM for 24 h as the sham treatment. In the co-culture nested system, chondrocytes were plated in the lower chamber of 6-well plates and divided into three groups as follows: control group, IL-1β group and Nanofat group. IL-1β group and Nanofat group were modeled by pre-treatment of IL-1β (10 ng/ml) for 24 h, and Nanofat cells were seeded in upper chamber (0.4-μm pore size; Corning) of 6-well plates of the Nanofat group for another 24-h treatment.

### Cell viability assay

The cell viability of chondrocytes was determined by CCK-8 assay at 24 and 48 h. Briefly, the chondrocytes were seeded on 96-well plates at a density of 5 × 10^3^ cells/well in 200 μl medium for 24 h, followed by the treatment of Nanofat-CM obtained from different seeding cell numbers (50, 100, 150, 200 × 10^4^ cells) for another 24 h and 48 h. Aliquots of each 20 μl CCK-8 solution were added to each well and incubated at 37 °C for 2 h, until the color turned to orange. The optical density (OD) value was measured at 450 nm with a microplate reader (Bio-Rad Laboratories, Inc., Hercules, CA, USA). Cell viability rate (%) = (Nanofat-CM-treated OD/untreated OD) × 100.

### Wound healing assay

Chondrocytes in the logarithmic growth phase were inoculated in 6-well plates at a density of 3 × 10^5^ cells per well and divided into three groups: control group, IL-1β group, and Nanofat-CM group. Before the scratch, IL-1β group and Nanofat-CM group were pre-treated with IL-1β for 24 h. Afterward, all groups were scratched using a sterile 200-μl pipette tip, followed by gently twice washes with PBS to remove cell debris. Subsequently, the Nanofat group was treated with Nanofat-CM for 0, 12 and 24 h. The cells were observed and imaged under an inverted microscope (CarlZeiss, Göttingen, Germany). The blank area (gap space) was estimated by Image J 1.47 software, and the ratio of the gap space at 12 h or 24 h to the gap space at 0 h was calculated for comparison. Each experiment was conducted in triplicate.

### Real-time PCR

The relative mRNA expression of targeted genes in chondrocytes was measured using a qPCR assay on an ABI QuantStudio™ 7 Flex Real-Time PCR System (Applied Biosystems; Thermo Fisher Scientific, Inc.). Total RNA of chondrocytes was extracted with TRIzol reagent and centrifuged at 12,000 rpm at 4 °C for 15 min. The purity and completeness of the samples were measured with NanoDrop2000 spectrophotometer (Thermo Scientific, USA), and the wavelength absorption ratio (260/280 nm) was around 2.0 for all samples. Then, the reverse transcription was carried out to obtain cDNA. As previously applied, the final qPCR reaction system was 20 μl, comprising 10 μl SYBR® Premix Ex Taq II (Tli RnaseH Plus), 0.4 μl PCR Forward Primer, 0.4 μl PCR Reverse Primer, 1 μl template cDNA and 8.2 μl ddH2O, and the qPCR reaction conditions included pre-incubation at 95 °C for 5 min, followed by 40 cycles of denaturation at 95 °C for 10 s, annealing and extension at 60 °C for 30 s. *β-Actin* was used as the reference gene, and 2^−ΔΔCT^ method was applied to measure the relative mRNA expression (Table 1).

**Table 1:** Self-designed primer sequences of target genes

Gene	Forward primer	Reverse primer
*β-Actin*	5′-CCCGCGAGTACAACCTTCT-3′	5′-CCCGCGAGTACAACCTTCT-3′
*Col2*	5′-CTCAAGTCGCTGAACAACCA-3′	5′-GTCTCCGCTCTTCCACTCTG-3′
*Col10*	5′-GATCATGGAGCTCACGGAAAA-3′	5′-CCGTTCGATTCCGCATTG-3′
*Aggrecan*	5′-GCAGACATTGATGAGTGCCTC-3′	5′-CTCACACAGGTCCCCTCTGT-3′
*Sox9*	5′-CATCAAGACGGAGCAACTGA-3′	5′-TGTAGTGCGGAAGGTTGAAG-3′
*IL-6*	5′-CTCTCCGCAAGTAAGTGAA-3′	5′-GGTATCCTCTGTGAAGTCTC-3′
*Adamts5*	5′-TGGAGTGTGTGGAGGGGATA-3′	5′-CGGACTTTTATGTGGGTTGC-3′
*Mmp3*	5′-TTGATGATGATGAACGATGG-3′	5′-CCTTCTTACCTCACTTCCTAT-3′
*Mmp13*	5′-CTATGGTCCAGGAGATGAAGAC-3′	5′-GTGCAGACGCCAGAAGAATCT-3′

### Western blot analysis

Total protein of chondrocytes was extracted with lysis buffer (50 mM Tris–HCl, pH 7.4, 150 mM NaCl, 1 mM EDTA, 1% Triton and 0.1% SDS) containing phosphatase and proteinase inhibitor cocktail (Bimake, Houston, TX, USA) for 30 min on ice. The protein concentration was assessed by a BCA kit (Thermo Fisher, USA). The targeted protein was separated by denaturing sodium dodecyl sulfate polyacrylamide gel electrophoresis (SDS-PAGE; 6–12%) and transferred to a nitrocellulose membrane (Sartorius Stedim, Göttingen, Germany). The membrane was blocked with 5% nonfat milk in Tris-buffered saline tween (TBST) at 4 °C for 2 h, which was followed by overnight incubation at 4 °C with the following primary antibodies against β-actin, Col2, Mmp9, Mmp13, and Sox9. Subsequently, the membranes were washed with TBST three times and incubated with peroxidase-conjugated goat antirabbit/mouse IgG at room temperature for 2 h; each protein was visualized using Western Lightning® Plus ECL (Perkin Elmer, Inc., Waltham, MA, USA), detected using X-ray film (Kodak, Tokyo, Japan) and scanned.

### Clinical retrospective study

This study was approved by the Ethics Committee of the First Affiliated Hospital of Zhejiang Chinese Medical University (Zhejiang Provincial Hospital of Chinese Medicine), number: 2019-X-001-01, and articular injection of Nanofat has been a routine medical treatment in the hospital. The data about 18 knee OA patients who have been treated with articular injection of autologous Nanofat in Zhejiang Provincial Hospital of Chinese Medicine were collected. The autologous Nanofat was derived from the abdominal subcutaneous fat tissue (about 30–40 ml) of each patient under anesthesia and was concentrated into 6 ml volume for each articular injection. Each joint was injected by Nanofat for only once. Informed consent has been obtained from all patients. For clinical evaluation of the Nanofat outcomes, visual analog scale (VAS), Western Ontario and McMaster Universities Osteoarthritis Index (WOMAC) and magnetic resonance imaging (MRI) examination were conducted 1 week before injection (baseline) and 9 months after injection. The inclusive criteria include: (1) age between 30 and 80 years old; (2) radiological and clinical diagnosis of OA; (3) grade 2 or more according to Kellgren–Lawrence criteria and pain intensity of grade 3 or more on VAS; and (4) at least two nonsurgical treatments have failed. The exclusive criteria include: (1) body mass index (BMI) ≥ 35; (2) patients who received additional knee joint operation or intra-articular injection of any drug during the follow-up period; (3) patients with rheumatoid arthritis, gout arthritis, severe traumatic arthritis and hemophiliac arthritis; (4) patients who have severe medical problems; (5) a history of allergies to any substance used in the treatment; and (6) pregnancy or became pregnant during treatment.

### Statistical analysis

SPSS13.0 software (SPSS, IL, USA) was used for data analysis. Data were expressed as mean values ± standard deviation (SD). The Student’s *t* test or one-way analysis of variance (ANOVA) was applied to evaluate the statistically significant difference between groups. Differences with a *P* value of < 0.05 were considered statistically significant.

## Results

### Characterization of Nanofat and ADSCs

**Fig. 1:**
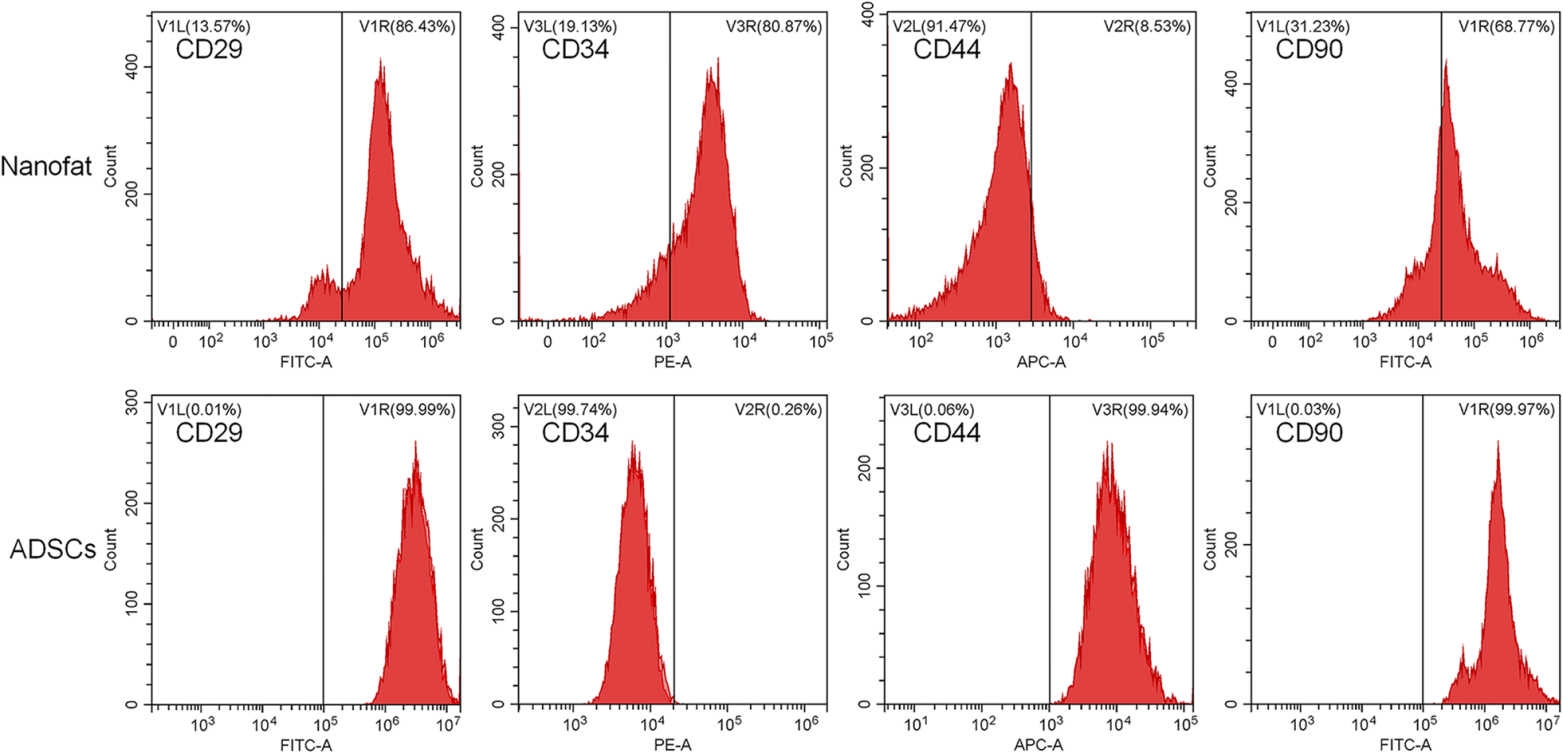
Flow cytometry analysis of Nanofat and ADSCs

As shown in Fig. 1, flow cytometry analysis showed that the uncultured Nanofat consisted of nearly 86.43% CD29^+^ cells, 80.87% CD34^+^ cells, 8.53% CD44^+^ cells and 68.77% CD90^+^ cells. And ADSCs accounted for more than 8.53% in Nanofat, based on the expression level of the CD44 marker, while cultured ADSCs showed high expression of CD29, CD44 and CD90 (all > 99%) and low expression of CD34 (< 1%). Nanofat expressed the classical ADSCs markers including CD29, CD44 and CD90, which was in line with another study [23, 30, 31], while the high expression of CD34, CD29 and CD90 could indicate the existence of endothelial progenitor cells, pericytes and vascular smooth muscle cells [23].

### Therapeutic effects of Nanofat and ADSCs on osteoarthritic rats

**Fig. 2:**
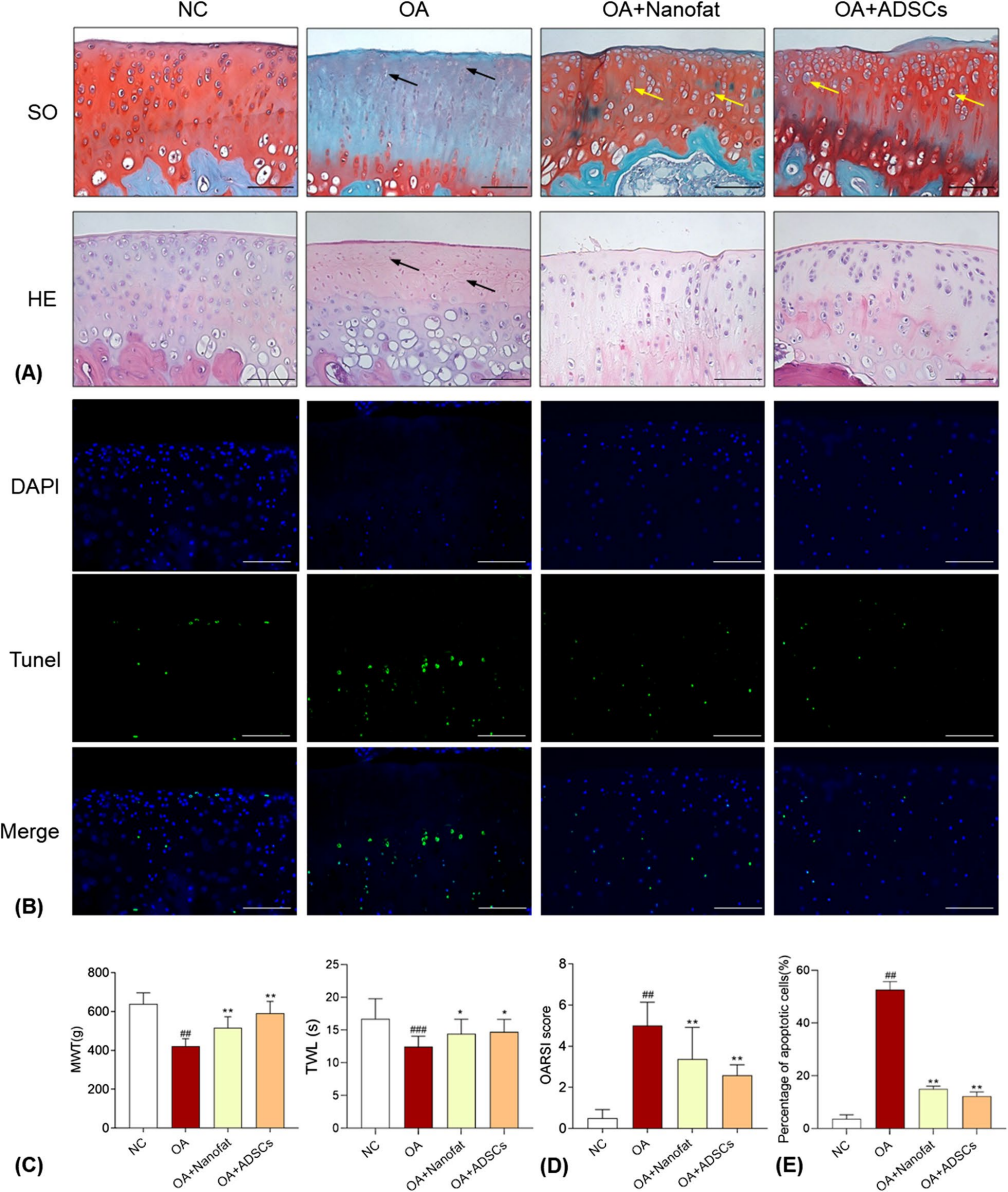
Effect of Nanofat and ADSCs on MIA-induced rat OA model. **A** Safranin O/Fast green (SO) staining, hematoxylin and eosin (H&E) staining with black arrows (loss of chondrocytes) and yellow arrows (hypertrophic chondrocytes). Scale bars = 100 μm; **B** TUNEL and DAPI staining of rat knee joints; **C** measurement of MWT and TWL of rats; **D** OARSI scores for histopathological observation; **E** percentage of apoptotic cells in TUNEL assay. Values were presented as mean ± SD. ^##^*P* < 0.01 versus NC group; *P < 0.05 and **P < 0.01 versus OA group

**Fig. 3:**
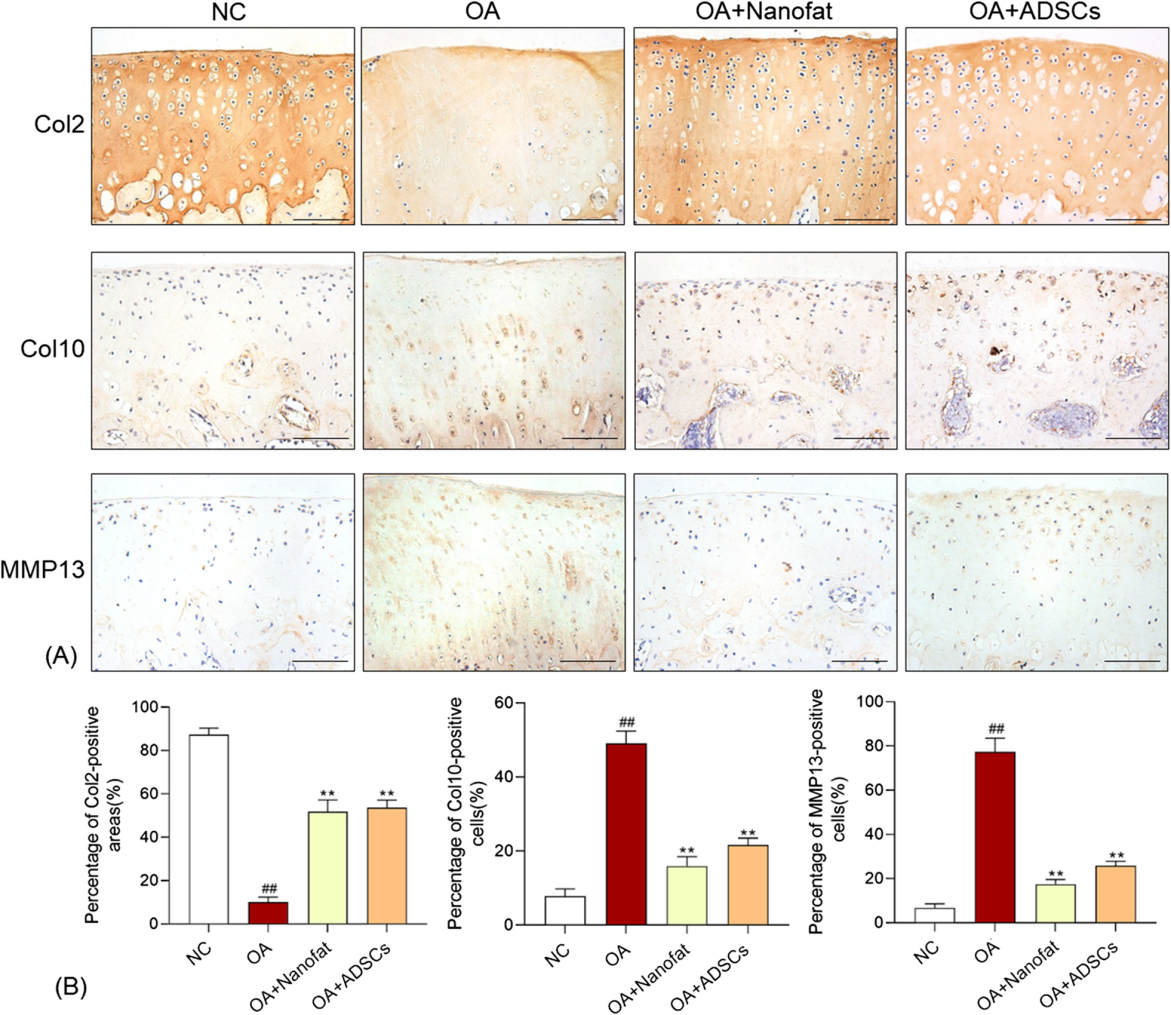
Effects of Nanofat and ADSCs on expressions of Col2, Col10 and MMP13 on rat cartilage. **A** Representative immunohistochemical staining of Col2, Col10 and MMP13 on cartilage; **B** quantitative measurement of positive area percentage of Col2 and positive cell percentages of Col10 and MMP13. Values were presented as mean ± SD. ^##^*P* < 0.01 versus NC group; **P < 0.01 versus model group

To study the potential effects of Nanofat and ADSCs on OA, we constructed a rat OA model by articular injection of MIA. After modeling, histopathological staining and TUNEL assay showed obvious cartilage degeneration with chondrocyte apoptosis and loss, collagen mass destruction, and matrix disorganization, whilst pain behavior assays showed abnormal allodynia and hyperalgesia, indicating a typical OA phenotype (Fig. 2A–C). As compared with the NC group, the number of chondrocytes and the content of glycosaminoglycan (the essential component of cartilage matrix) were remarkably reduced and the apoptotic chondrocytes were significantly increased in the model group (*P* < 0.01) (Fig. 2A, B, E), with significantly increased OARSI score (*P* < 0.01) (Fig. 2D). Moreover, the mechanical allodynia (reflected by MWT) and thermal hyperalgesia (reflected by TWL) were significantly decreased in the model group (*P* < 0.01 vs. NC) (Fig. 2C). By contrast, intra-articular injection of Nanofat and ADSCs significantly increased the number of chondrocytes, decreased the apoptotic chondrocytes, improved the structural integrity and glycosaminoglycan synthesis of articular cartilage, and relieved the mechanical allodynia and thermal hyperalgesia in the Nanofat and ADSCs groups, with significantly decreased OARSI score (each *P* < 0.01 vs. model) (Fig. 2A–E). There remained some hypertrophic chondrocytes, characterized by a more round shape and a large intracellular space, in the cartilage of Nanofat and ADSCs groups (Fig. 2A). Immunohistochemical results indicated that Col2 (principal component of cartilage matrix), Col10 (hypertrophic marker) and MMP13 (catabolic marker) were significantly altered in the model group (each *P* < 0.01 vs. NC) (Fig. 3). After the treatment with Nanofat and ADSCs, the expressions of those markers were significantly restored to the normal levels (each *P* < 0.01 vs. NC) (Fig. 3). Notably, there was no significant difference between the Nanofat group and ADSCs group in terms of histopathological OARSI scores, TUNEL staining, and pain behavior, suggesting that Nanofat and ADSCs had comparable therapeutic efficacy in OA rats.

### Nanofat-CM promoted the cell viability of chondrocytes

**Fig. 4:**
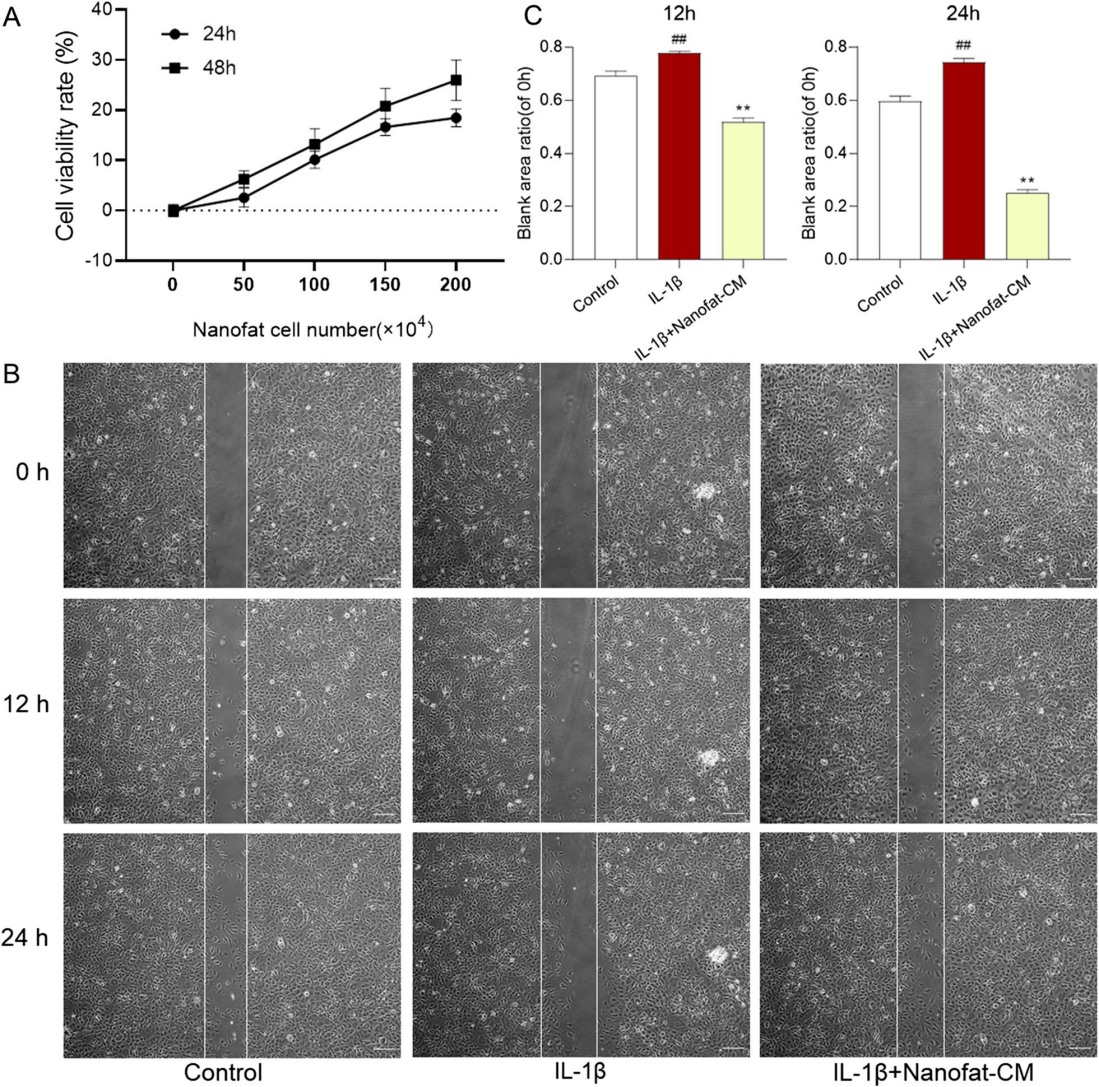
Effects of Nanofat-CM on cell viability and wound healing ability of chondrocytes. **A** Cell viability assay at 24 h and 48 h after Nanofat-CM treatment; **B** Wound healing assay of chondrocytes with Nanofat-CM treatment at 0 h, 12 h and 24 h, scale bars = 100 μm; **C** quantification of blank area ratio (of 0 h) at 12 h and 24 h. Values were presented as mean ± SD. ^##^*P* < 0.01 versus control group; **P < 0.01 versus IL-1β group

To detect the effects of Nanofat-CM on the cell viability of chondrocytes, chondrocytes were cultured with Nanofat-CM. The results of CCK-8 assay showed that Nanofat-CM derived from different cell densities significantly increased the cell viability of chondrocytes after 24- and 48-h treatment (Fig. 4A).

### Nanofat-CM induced wound healing of IL-1β-treated chondrocytes

Nanofat consists of various cell types and may have paracrine-dependent benefits toward chondrocytes through the secretome in Nanofat-CM. Therefore, a wound healing assay was conducted to analyze the wound healing effect of Nanofat-CM on IL-1β-treated chondrocytes. Chondrocytes treated with IL-1β exhibited almost no wound healing capacity, whereas the ratio of wound area with Nanofat-CM treatment at 12 and 24 h to the area without treatment (0 h) was significantly decreased, indicating that the addition of Nanofat-CM significantly induced the migration of IL-1β-treated chondrocytes (Fig. 4B, C).

### Nanofat and Nanofat-CM reversed the abnormal gene expression of IL-1β-treated chondrocytes

**Fig. 5:**
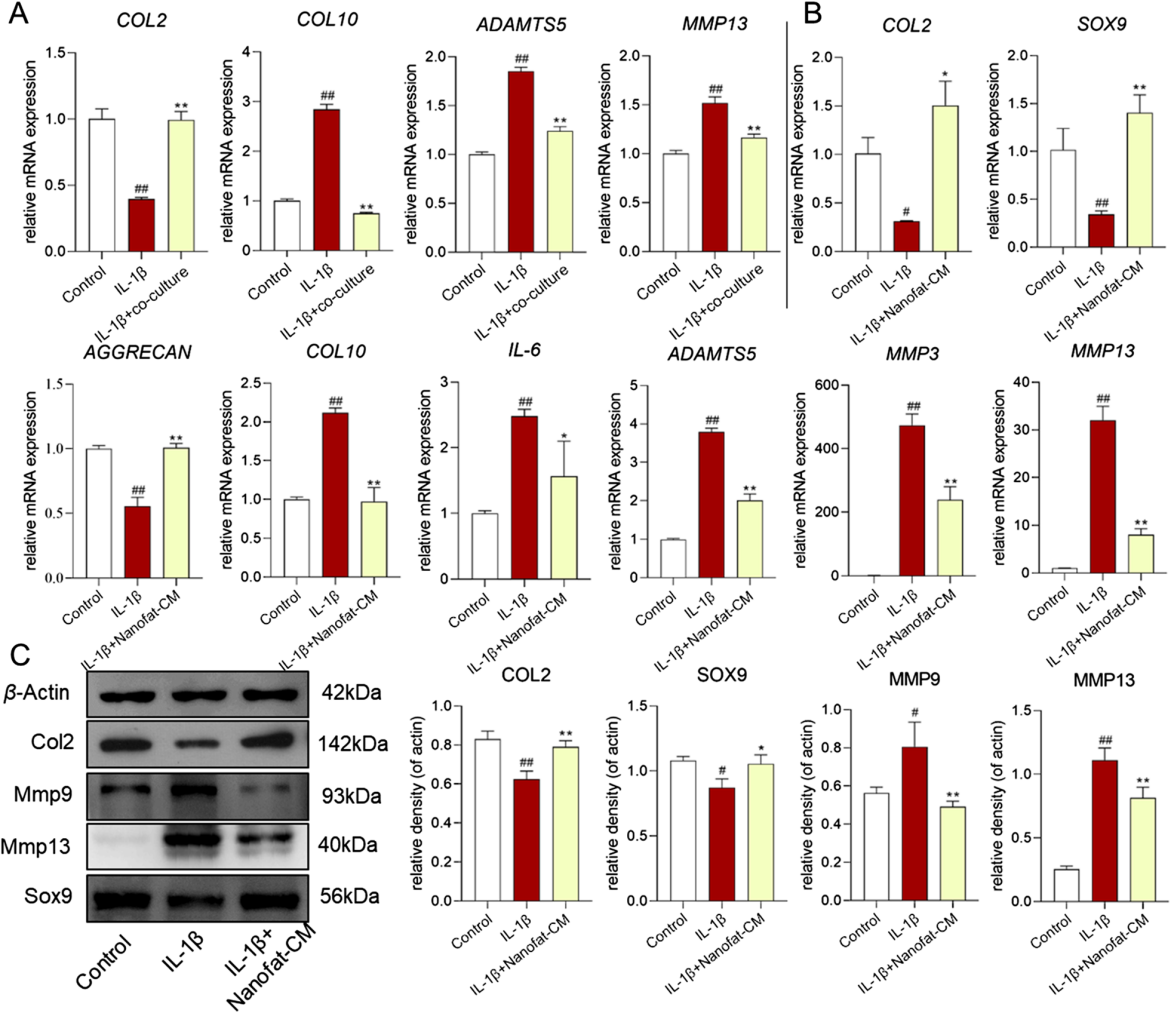
Regulations of Nanofat and Nanofat-CM on IL-1β-treated chondrocytes. **A** qPCR analysis of mRNA expressions of chondrocytes in co-culture with Nanofat; **B** qPCR analysis of mRNA expressions of chondrocytes treated with Nanofat-CM; **C** expressions of target proteins in chondrocytes treated with Nanofat-CM. Values were presented as mean ± SD. ^#^*P* < 0.05 and ^##^*P* < 0.01 versus control group; *P < 0.05 and **P < 0.01 versus IL-1β group

To evaluate the effect of Nanofat on IL-1β-induced chondrocytes, we established a co-culture system using a six-well plate and cell culture inserts that included polyethylene terephthalate (PET) membrane with 0.4-μM pores. Our results suggested that the decreased mRNA expression of *Col2* and the increased mRNA expression of *Col10*, *Adamts5* and *Mmp13* induced by IL-1β in chondrocytes, were significantly reversed in the co-culture group (Fig. 5A).

To investigate the protective effects of Nanofat-CM on IL-1β-induced chondrocytes, the cells were cultured with 10 ng/ml IL-1β for 24 h followed by treatment with Nanofat-CM for another 24 h. We first performed qPCR to examine the regulative effects of Nanofat-CM on gene expressions in rat chondrocytes. The qPCR results showed that IL-1β significantly increased *Col10*, *Adamts4*, *Adamts5*,* Mmp3*, *Mmp13* mRNA expression and decreased *Col2*, *Aggrecan*,* Sox9* mRNA expression in rat chondrocytes. Such altered gene expressions were significantly reversed by Nanofat-CM after 24-h treatment (Fig. 5B). Next, we examined the effects of Nanofat-CM on IL-1β-induced chondrocytes by western blot. Consistent with our qPCR results, western blot indicated that Nanofat-CM significantly reversed the expression of Col2, Sox9, Mmp9, Mmp13 protein in chondrocytes induced by IL-1β (Fig. 5C). Taken together, the above results indicated that Nanofat and Nanofat-CM could effectively reverse the abnormal gene expression changes induced by IL-1β in rat chondrocytes.

### Intra-articular Nanofat injections alleviated symptoms and pain in OA patients

**Fig. 6:**
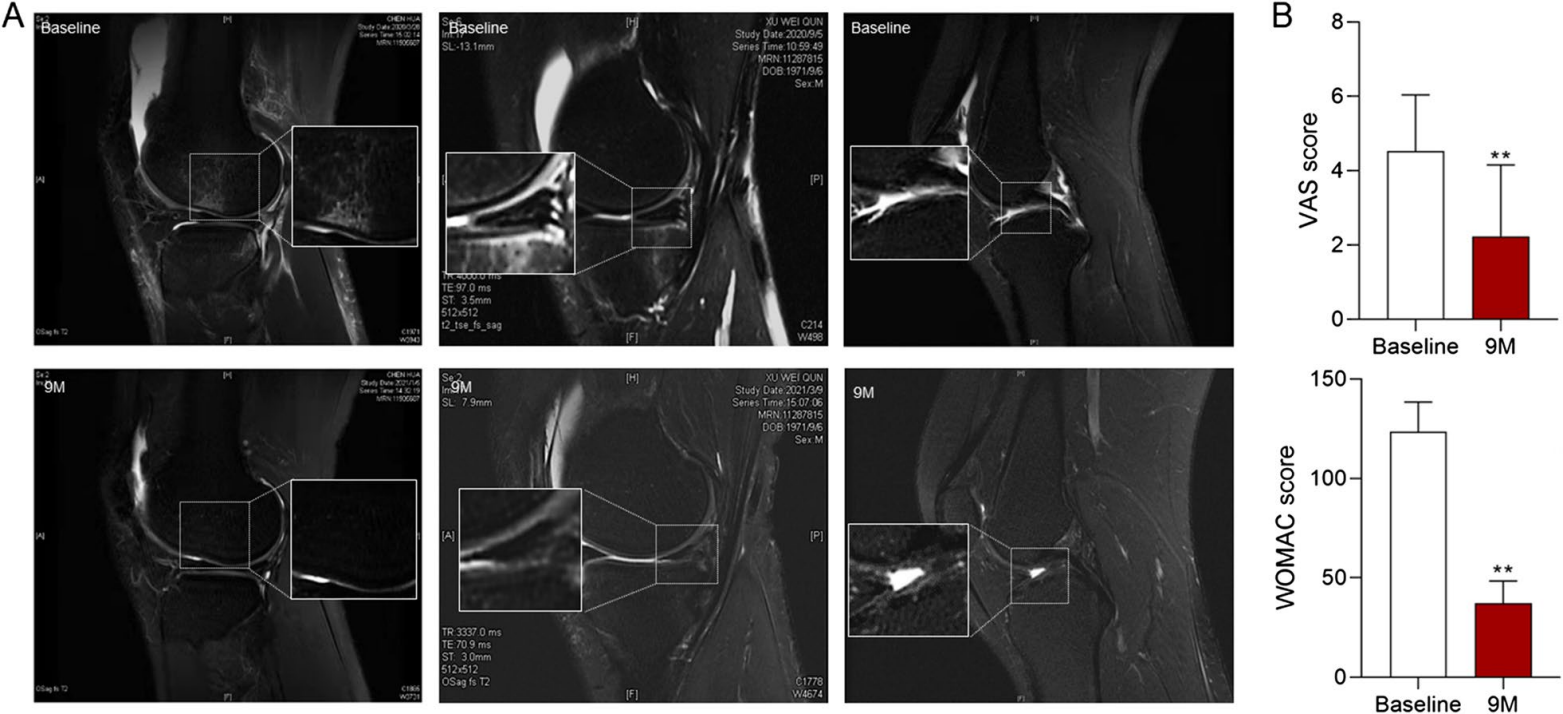
Imaging analysis and OA scoring of clinical subjects. **A** Magnetic resonance imaging observation; **B** WOMAC, VAS scores of patients with knee OA before (baseline) and after once intra-articular injection of Nanofat for 9 months (9 M). Values were presented as mean ± SD. **P < 0.01 versus baseline group

We conducted a clinical retrospective study to investigate the efficacy of intra-articular autologous Nanofat injections and to assess the effects of Nanofat injections on articular cartilage. The patients showed no significant difference in age, BMI and injection dose between different gender (Table 2). No major adverse events related to the injections were observed during the treatment and follow-up periods, except for 1 case, in which the patient experienced marked pain with swelling after the injection, which resolved spontaneously after 1 week. The VAS score and WOMAC score decreased by 50.8% and 69.8%, respectively, which indicated a statistically significant improvement after 9 months post-injection versus baseline measures (Fig. 6B). For MRI review, three patients completed MRI evaluation 9 months after Nanofat injection. MRI results showed that after 9 months of treatment, the patient’s bone marrow edema had disappeared, the joint effusion had decreased, and the articular cartilage and meniscus damage had been repaired to varying degrees (Fig. 6A).

**Table 2:** Clinical information of OA patients received Nanofat injection

	Age (years)	BMI (kg/m^2^)	Left	Right	Both	Injection volume (ml)
Male (*n* = 7)	55.57 ± 13.69	24.03 ± 2.35	2	3	2	9.11 ± 1.83
Female (*n* = 11)	56.09 ± 13.43	24.87 ± 2.97	3	3	5	9.97 ± 3.05
*P* value	0.94	0.54	–	–	–	0.45

## Discussion

Although Nanofat has been attempted for treating OA in clinic [32], its efficacy and mode of action remain poorly understood. To bridge this gap, the present study evaluated the in vivo anti-OA effects of Nanofat by using MIA-induced OA rat model and recruiting OA patients and explored the cellular and molecular mode of action of Nanofat by applying IL-1β-induced model of chondrocytes. The in vivo data demonstrated that Nanofat exerted multiple effects against OA through ameliorating joint pain and protecting chondrocytes and cartilage ECM from damage (Fig. 2). Moreover, the anti-OA effects were not significantly different between Nanofat and ADSCs (*P* > 0.05), indicating that Nanofat could replace ADSCs for OA treatment (Fig. 1). The in vitro data demonstrated that Nanofat exerted protective effects on chondrocytes through restoration of anabolic metabolism (up-regulation of Col2, Aggrecan, and Sox9), inhibition of catabolic metabolism (down-regulation of Adamts5, Mmp3, Mmp9 and Mmp13), inhibition of inflammation (down-regulation of IL-6), and suppression of hypertrophy (down-regulation of Col10) (Fig. 4). Moreover, the regulatory actions on anabolic, catabolic, and hypertrophic genes on chondrocytes were similar between co-cultured Nanofat and Nanofat-CM, suggesting a paracrine-based mode of action of Nanofat. The main innovation and characteristic points of this study are: (1) both the experimental and clinical determination of anti-OA efficacy and safety of Nanofat; (2) the comparison of therapeutic effect between Nanofat and ADSCs; and (3) the exploration of paracrine-based action of Nanofat by using its conditioned medium.

Nanofat is a heterogeneous mixture of cells that includes preadipocytes, EPCs, vascular adventitial cells, pericytes, macrophages, and MSCs. These cell types have been shown to exert promising effects in tissue regeneration. The preadipocytes express some similar phenotypic markers and characteristics to MSCs and thereby possess regenerative capacity as MSCs [33]. The EPCs and vascular adventitial cells are capable of promoting angiogenesis and neovascularization, and the pericytes can improve revascularization and regenerate muscle fibers [34]. The macrophages derived from adipose possess anti-inflammatory activity with an immunosuppressive phenotype (M2 phenotype) [35]. The pericytes can improve revascularization and regenerate muscle fibers [36]. MSCs in adipose are ADSCs that play an important role in tissue regeneration and act as a major cell component of Nanofat [37]. Co-culture of chondrocytes with ADSCs resulted in decreased production of inflammatory mediators [38]. Moreover, the soluble factors secreted from ADSCs promoted cell proliferation and inhibit apoptosis of chondrocytes [39]. However, the anti-OA efficacy of Nanofat was not only relying on ADSCs in the present study, indicating a combined or synergistic mode of action of a variety of cells in Nanofat. Furthermore, we demonstrated that paracrine action carried out the main role of Nanofat in treating OA. Many cell components in Nanofat have paracrine capacities, including macrophages, EPCs, pericytes, fibroblasts, stromal cells, and ADSCs. Macrophages secrete receptor antagonists of IL-1 and IL-10 to exert immunosuppressive effects [40]. EPCs release many growth factors (vascular endothelial growth factor and insulin-like growth factor-1) to exert regenerative effects [40, 41]. Pericytes participate in immune and inflammatory responses by producing cytokines in response to pathological stimuli [42]. Fibroblasts secrete extracellular matrix proteins and growth factors that may contribute to regenerate [43, 44]. Stromal cells secrete extracellular matrix components that enhance the general capacity for cell adhesion, migration, cell–matrix interaction, and regeneration [45]. ADSCs also secrete a large number of cytokines, growth factors, and exosomes which function as immunomodulators, trophic, antiapoptotic, anti-fibrosis, and angiogenic factors [46].

Nanofat and ADSCs are homologous biomaterials derived from adipose tissue. ADSCs have been reported to exert chondroprotective efficacy against OA. In clinic, ADSCs, no matter allogeneic or autologous, can relieve pain and improve cartilage regeneration in OA patients [47]. However, the therapeutic efficacy of ADSCs is relying on a large cell number, which requires a large-scale and long-term ex vivo expansion with increasing risks of contamination and allergy to heterologous substances as well as unexpected cell differentiation [48]. A 2-year follow-up study reported that high dose of ADSCs (1 × 10^8^ cells) significantly improved the symptoms of OA patients, but ADSCs at medium (5 × 10^7^ cells) and low doses (1 × 10^7^ cells) resulted in worsening outcomes after 1 year [49]. Moreover, clinical application of culture-expanded stem cells (including ADSCs) is commonly restricted by ethical concerns in many countries [21]. The above shortcomings of ADSCs have limited the development of ADSCs-based therapies. Comparatively, Nanofat overcomes the shortcomings of ADSCs, since it has no need of ex vivo expansion and can be immediately processed and directly used at the bedside. Therefore, Nanofat is more feasible and applicable for clinical applications. According to our clinical retrospective analysis, single articular injection of Nanofat significantly restored the pain-related scores (VAS and WOMAC) and repaired the cartilage and meniscus damage and bone marrow edema of patients after 9 months (*P* < 0.05). More importantly, no adverse event was observed with Nanofat treatment during the follow-up observation. Consequently, Nanofat is a safe and efficient cell therapy that has advantages over ADSCs for the treatment of OA.

## Conclusion

This study demonstrated that Nanofat exerted anti-OA efficacy by ameliorating joint pain symptoms and preventing cartilage degradation of OA rats through paracrine-based actions on anabolic, catabolic, and hypertrophic molecules of chondrocytes. A retrospective study further verified the clinical efficacy and safety of Nanofat. Many cell types, including ADSCs, might secrete bioactive factors and contribute to the beneficial effects of Nanofat, but the concrete roles of each cell type in Nanofat remain unclear, warranting further investigations. Particularly, it can be confirmed that the anti-OA efficacy of Nanofat was similar to that of ADSCs. Since the preparation of Nanofat is more convenient with less ethical restriction and less safety concern than ADSCs, Nanofat can thereby be rapidly applied in clinic as a superior and practical substitute of ADSCs for OA treatment. In sum, this study provides novel knowledge of Nanofat’s anti-OA efficacy and paracrine-based action of mode on OA, suggesting it as a promising and practical cell therapy for clinical treatment of OA.

## Data Availability

The datasets used and/or analyzed during the current study are available from the corresponding author on reasonable request.

## Abbreviations

OA: Osteoarthritis
MIA: Monoiodoacetate
ADSCs: Adipose tissue-derived stem cells
NSAIDs: Nonsteroidal anti-inflammatory drugs
PRP: Platelet-rich plasma
MSCs: Mesenchymal stem cells
PDGF: Platelet-derived growth factor
TGF-β: Tumor-like growth factor-β
FGF: Fibroblast growth factor
VEGF: Vascular endothelial growth factor
HGF: Hepatocyte growth factor
IGF-1: Insulin-like growth factor-1
EPCs: Endothelial progenitor cells
CM: Conditioned medium
α-MEM: Minimum essential medium-alpha modification
IMDM: Iscove’s modified Dulbecco’s medium
FBS: Fetal bovine serum
PCR: Polymerase chain reaction
HBSS: Hank’s balanced salt solution
FITC: Fluorescein isothiocyanate
PE: Phycoerythrin
APC: Allophycocyanin
SD: Sprague–Dawley
MWT: Mechanical withdrawal threshold
TWL: Thermal withdrawal latency
HE: Hematoxylin and eosin
SO: Safranin O/Fast green
Col2: Collagenase type II
TBST: Tris-buffered saline tween
VAS: Visual analog scale
WOMAC: Western Ontario and McMaster Universities Osteoarthritis Index
MRI: Magnetic resonance imaging
BMI: Body mass index
